# Dual-Model Synergy for Fingerprint Spoof Detection Using VGG16 and ResNet50

**DOI:** 10.3390/jimaging11020042

**Published:** 2025-02-04

**Authors:** Mohamed Cheniti, Zahid Akhtar, Praveen Kumar Chandaliya

**Affiliations:** 1 Faculty of Electrical Engineering, Telecommunications Department, Laboratory (LTIR), University of Science and Technology Houari Boumediene, BP.32, EI-Alia, Bab-Ezzouar, Algiers 16111, Algeria; 2 Department of Network and Computer Security, State University of New York Polytechnic Institute, Utica, NY 13502, USA; akhtarz@sunypoly.edu; 3 Department of Artificial Intelligence Sardar Vallabhbhai National Institute of Technology, Surat 395007, Gujarat, India; pkc@aid.svnit.ac.in

**Keywords:** fingerprint liveness detection, VGG16, ResNet50, Livedet2013, Livedet2015, spoof attacks, error rate analysis (BPCER, APCER), biometric anti-spoofing

## Abstract

In this paper, we address the challenge of fingerprint liveness detection by proposing a dual pre-trained model approach that combines VGG16 and ResNet50 architectures. While existing methods often rely on a single feature extraction model, they may struggle with generalization across diverse spoofing materials and sensor types. To overcome this limitation, our approach leverages the high-resolution feature extraction of VGG16 and the deep layer architecture of ResNet50 to capture a more comprehensive range of features for improved spoof detection. The proposed approach integrates these two models by concatenating their extracted features, which are then used to classify the captured fingerprint as live or spoofed. Evaluated on the Livedet2013 and Livedet2015 datasets, our method achieves state-of-the-art performance, with an accuracy of 99.72% on Livedet2013, surpassing existing methods like the Gram model (98.95%) and Pre-trained CNN (98.45%). On Livedet2015, our method achieves an average accuracy of 96.32%, outperforming several state-of-the-art models, including CNN (95.27%) and LivDet 2015 (95.39%). Error rate analysis reveals consistently low Bonafide Presentation Classification Error Rate (BPCER) scores with 0.28% on LivDet 2013 and 1.45% on LivDet 2015. Similarly, the Attack Presentation Classification Error Rate (APCER) remains low at 0.35% on LivDet 2013 and 3.68% on LivDet 2015. However, higher APCER values are observed for unknown spoof materials, particularly in the Crossmatch subset of Livedet2015, where the APCER rises to 8.12%. These findings highlight the robustness and adaptability of our simple dual-model framework while identifying areas for further optimization in handling unseen spoof materials.

## 1. Introduction

Biometrics systems, particularly fingerprint recognition systems, are widely used in various fields, from mobile devices to national security, due to their reliability, ease of use, and affordability [1,2]. However, as these systems have become more common, they have also become targets for security breaches, particularly via spoofing attacks. These attacks involve creating fake fingerprints from materials like silicone, gelatin, or even using advanced 3D printing techniques to mimic real fingerprints and trick biometric systems [3]. As a result, ensuring the security of fingerprint-based systems has become a critical challenge. To counter these threats, researchers are developing advanced methods like ‘liveness detection’ that distinguishes between genuine fingerprints and artificial replicas. Such countermeasure frameworks analyze deeper and multi-level characteristics to improve the robustness of fingerprint authentication systems [4]. Liveness detection methods for fingerprints can be categorized into two types, i.e., hardware-based and software-based [5]. Hardware-based techniques use extra devices to assess whether the fingerprint originates from a living person. Hard-ware-based techniques analyze physiological factors such as temperature, blood pressure, and pulse. While these methods can effectively distinguish between real and fake finger-prints, they add complexity and cost to the system. Moreover, adapting to new, more sophisticated spoofing techniques can be challenging. Software-based techniques have gained popularity, which rely on image/signal processing to extract characteristics from fingerprint samples. Unlike hardware solutions, software approaches are less costly and easier to upgrade when needed [6].

In recent years, deep learning-based fingerprint detection has garnered more attention. In particular, Convolutional Neural Networks (CNNs) have shown astounding performance in a variety of fields, including image and biometric spoof classification. The CNNs are very useful for pattern recognition applications and fingerprint liveness detection owing to their hierarchical features that can be automatically extracted. For instance, the study in [7] employed a pre-trained CNN to differentiate between fake and real fingerprints. The system was evaluated using datasets from the Liveness Detection Competitions held in 2009, 2011, and 2013. While achieving an impressive overall accuracy of 95.5% and first place in the LivDet2015 competition, the approach faced challenges in handling unknown spoof materials that often degraded performance. Similarly, Park et al. [8] developed a patch-based method for detecting fake fingerprints using a fully CNN with minimal parameters and an optimized threshold. This method achieved an average classification error of 1.35%. Despite its high accuracy, the method’s reliance on patch-based processing increased computational complexity and made it less scalable for larger datasets. Another study [9] utilized CNNs to extract features from fingerprint patches and achieved a classification error rate of 3.42% on the LivDet2009 dataset. However, this method struggled to generalize across datasets with different sensor types or spoofing materials. Additionally, a CNN with contrast enhancement was devised in [10] for fingerprint spoof detection, which achieved an impressive average accuracy of 99.8% on the ATVS database, but the dependency on dataset-specific pre-processing techniques limited its generalization to other datasets. Another work in [11] proposed a CNN and GAN-based approach using an Open Patch Generator (OPG) to create realistic spoof samples. It achieved high accuracy on LivDet databases (i.e., 96.20%, 94.97%, and 92.90% for 2015, 2017, and 2019, respectively) and robustness in cross-material and cross-sensor scenarios. Similarly, ref. [12] introduced a dynamic ensemble method combining deep CNN and handcrafted features. This framework obtained accuracies of 96.10%, 96.49%, and 94.99% on LivDet 2015, 2017, and 2019 databases, respectively, and outperformed state-of-the-art methods by integrating deep learning with traditional techniques. Also, ref. [13] proposed a Siamese attention residual CNN (Res-CNN) to exploit ridge continuity features (RCFs) and utilized Gabor filters, ridge continuity amplification loss, and transfer learning to attain superior performance in cross-material and cross-sensor experiments, validated through interpretable heatmap visualizations. While the work in [14] presented a Fisher vector learning-based method that combined spatial and frequency domain features, including local and global Fourier transforms, this technique reduced classification errors to 5.16%, 1.40%, and 7.51% on LivDet 2011, 2013, and 2015 databases, respectively. Collectively, these approaches highlight the effectiveness of combining advanced deep learning techniques with innovative feature extraction methods to enhance fingerprint liveness detection in real-world applications. Furthermore, ref. [15] further improved liveness detection by using noise analysis and textural pattern differences. It achieved 99.52% accuracy utilizing an ensemble classifier with multi-objective genetic algorithms and entropy-based features, surpassing classifiers like quadratic SVM (97.34%). Although CNNs have demonstrated encouraging outcomes in the identification of live fingerprints, significant challenges remain in achieving high accuracy, robustness, and generalization under varying types of fake fingerprints. One promising approach is the deep learning-based ensemble methods. Namely, multi-stream CNN-based fingerprint spoof detection frameworks, e.g., a single model architecture that combines two pre-trained CNNs, such as VGG16 and ResNet50 [7,16]. Multi-stream frameworks have demonstrated their effectiveness across various applications. Consequently, this study examined the performance of CNN-based multi-stream frameworks in fingerprint spoof detection. The primary contributions of this paper are outlined below.

1. Novel Dual-Model Framework: We propose the dual-model framework in fingerprint presentation attack detection (PAD) that combines VGG16 and ResNet50 architectures. This innovative approach employs the complementary strengths of both models (VGG16’s high-resolution feature extraction and ResNet50’s deep feature learning) to achieve superior generalization across diverse spoofing materials and sensor types. This contribution is significant because it is simple but yet addresses the limitations of single-model approaches that often struggle with variability in spoofing materials and sensor conditions.
2. Enhanced Feature Representation: By concatenating features from VGG16 and ResNet50, our framework creates a more robust and comprehensive representation of fingerprint data. This fusion enables the model to capture both fine-grained details (via VGG16) and high-level abstract features (via ResNet50), leading to improved discrimination between live and spoofed fingerprints. This represents a clear advancement over existing methods that rely on single-model feature extraction, as demonstrated by our state-of-the-art results on the LivDet2013 and LivDet2015 datasets.
3. State-of-the-Art Performance: Our framework achieves 99.72% accuracy on LivDet2013 and 96.32% accuracy on LivDet2015, outperforming several existing methods, including Gram model, Pretrained CNN, and CNN. Moreover, our devised framework achieves consistently low error rates, with a BPCER of 0.28% on LivDet2013 and 1.45% on LivDet2015, and an APCER of 0.35% on LivDet2013 and 3.68% on LivDet2015. These results demonstrate the practical effectiveness of our approach and its potential for real-world deployment in biometric security systems.
4. Robustness to Unseen Spoof Materials: While our framework shows strong performance across known spoofing materials, we also identify areas for improvement, particularly in handling unseen spoof materials (e.g., higher APCER in the Crossmatch subset of LivDet2015). The analysis provides valuable insights for future research in improving generalization to unknown attack scenarios. This contribution highlights both the strengths and limitations of our approach, offering a clear direction for further optimization.

The rest of this paper is structured as follows: Section 2 details the design and functionality of the proposed architecture. Databases, performance metrics used for evaluation, and implementation are discussed in Section 3. Section 4 presents experimental results and comparative analysis. Finally, conclusions are drawn in Section 5.

## 2. Proposed Dual-Stream Fingerprint Presentation Detection Framework

**Figure 1:**
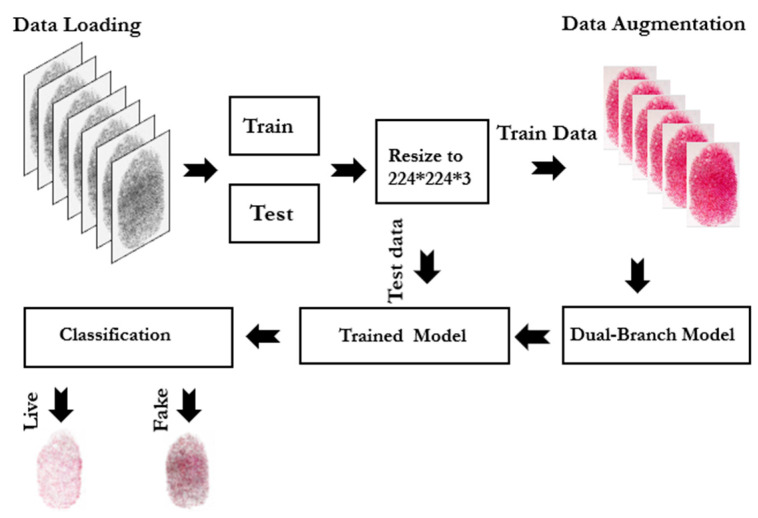
Proposed framework for fingerprint spoofing attack.

Our proposed approach leverages transfer learning by fine-tuning two well-established pre-trained models, i.e., VGG16 and ResNet50. Both models have demonstrated remarkable performances in various image identification applications [17]. These models were initially trained on the ImageNet dataset [18], which contains millions of labeled images. Thereby, these models are equipped to extract rich and discriminative characteristics of the input data. The selection of VGG16 and ResNet50 was driven by their proven ability to handle diverse and complicated real and fake fingerprint samples. Moreover, these models can sufficiently recognize fine details like ridges, valleys, and texture differences, which are essential for fingerprint detection either live or fake. Figure 1 depicts the proposed approach to detect fingerprint spoofing attacks.

The proposed approach involves five key steps. The data acquisition stage involves capturing the input fingerprint (irrespective of live or fake) under controlled conditions to highlight differences in texture and gray-level patterns. Genuine fingerprints show natural ridge and skin texture variations. The spoofed fingerprints often lack these details, reflecting the spoofing material. The preprocessing stage retains images in their original grayscale format to preserve critical features without binarization. The augmentation stage applies techniques like rotation, scaling, and brightness adjustments uniformly to both genuine and spoofed samples, which leads to enhancements of dataset diversity and model robustness. This approach ensures the model generalizes across different conditions without altering the core distinguishing features of live and spoofed fingerprints. The feature extraction stage uses fine-tuned pre-trained CNN models (i.e., VGG16 and ResNet50) to capture high-level features like texture and ridge flow. Finally, the classification stage uses a fully connected ANN to differentiate live from spoofed fingerprints. More details of these stages are described in the following subsections.

### 2.1. Data Preprocessing

Before applying transfer learning, several operations were performed to input data. This included resizing images to match the input size expected by VGG16 and Res-Net50 (224 × 224), normalizing pixel values to the range [0, 1], and applying data augmentation, i.e., performing several transformations like artificially increasing the size and diversity of our training dataset. This reduces overfitting and increases the robustness of the model, especially when using a small dataset. A variety of image augmentation techniques were used. Specifically, the images are randomly rotated between −20 to +20 degrees. Also, width and height shifts are used that involve shifting an image horizontally or vertically by a certain percentage of its dimensions. Consequently, this helps our model to handle variation in the position of the input fingerprint images [19]. To increase the resilience of the model, shear, zoom, and flip augmentations are methods that help to produce several image versions. The training data are augmented for diversity via shear (tilting the image along an axis), zoom (adjusting the image’s scale), and flipping (altering the image’s orientation). However, when applying transformations like rotation, shift, or zoom, some pixels in the image may appear vacant or absent. Therefore, the gaps of augmentation addresses are filled using techniques like nearest-neighbor interpolation, thus ensuring the image remains complete for training [20]. Additionally, testing data are not augmented, as they are used to evaluate the model’s performance on unaltered, real-world-like data.

### 2.2. Feature Extraction

After resizing and applying different augmentation techniques, the input sample is sent to dual pre-trained CNN models, i.e., VGG16 and Resnet50. However, the adopted models are used without their final fully connected (classification) layers, which are re-sponsible for making predictions based on the features extracted from the earlier layers. It means that the final classification layers (which are usually used for the ImageNet classes) are detached. Furthermore, removing the top layer lets the remaining parts of the models (composed of convolutional and pooling layers) serve as powerful feature ex-tractors [7]. First, the fingerprint images are fed into the modified models; as the pictures of the fingerprint pass through the convolutional layers, each layer extracts gradually intricate features. The lower layers might capture elementary patterns (like edges), while the deeper layers capture more abstract features (like shapes and forms specific to fingerprints).

### 2.3. Feature Concatenation

Feature concatenation refers to the process of combining multiple feature vectors (or tensors) from various models or layers into a single, cohesive feature vector. Rather than using arithmetic operations like addition or multiplication, this approach simply stacks the features side by side. After the input image has passed through all layers of both Res-Net50 and VGG16, we take the outputs of the final layers (before their respective classification heads) and flatten them into 1D vectors. VGG16 outputs a feature map with dimensions (7, 7, 512) for each image, while ResNet50 outputs a feature map with dimensions (7, 7, 2048). The flattened vector for VGG16 results in 25,088 features, and the flattened vector for ResNet50 results in 100,352 features. By concatenating these feature outputs, we create a larger feature vector that contains both detailed texture information from VGG16 and deeper, more complex patterns from ResNet50. This gives the model a richer set of data to work with, often improving its ability to distinguish between real and spoofed fingerprints. For ResNet50 and VGG16, concatenation is a better choice because of the following:

- These networks capture complementary features (ResNet50 excels at abstract, high-level features, while VGG16 specializes in simpler, low-level features).
- We want to preserve the distinct information each network provides, rather than blending it together through addition.
- Concatenation results in a larger and more diverse feature space, which can help the final classifier make more informed decisions.

### 2.4. Model Building

**Figure 2:**
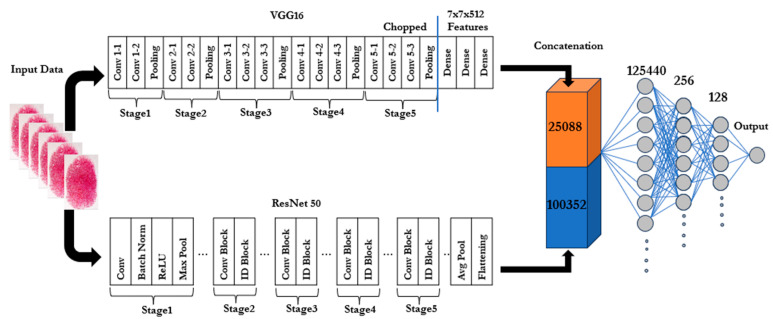
Architecture of the dual-branch proposed model for fingerprint liveness detection.

Following the extraction and flattening of the features from the VGG16 and ResNet50 models, Artificial Neural Network (ANN) was used to classify our input data. The input layer, hidden layers, and output layer are the three layers that make up the ANN. With its input dimension equal to the number of combined features (a high number coming from flattening both feature maps), the input layer receives the concatenated features from VGG16 and ResNet50. The first hidden layer is made up of 256 neurons with ReLU activation. This is intended to record intricate feature correlations. The learnt representations are further refined by the 128 neurons in the second hidden layer that are likewise activated by ReLU. The output layer (the last layer) is made up of a single neuron that has a sigmoid activation function and is in charge of binary classification (spoof vs. live). Adam (Adaptive Moment Estimation) is the optimizer in deep learning. It minimizes the binary cross-entropy loss during training. By combining the benefits of AdaGrad (Adaptive Gradient Algorithm) and RMSProp (Root Mean Square Propagation) Adam offers efficient and adaptive optimization. This makes it ideal for issues involving sparse gradients or big datasets. Additional details are illustrated in Figure 2, and all the steps are summarized in Algorithm 1.
**Algorithm 1: Fingerprint Liveness Detection Using Dual Pre-Trained Models**1: Procedure2: Input    - X_train, Y_train: Training dataset and labels. - VGG16(·): Pre-trained VGG16 model for high-resolution feature extraction. - ResNet50(·): Pre-trained ResNet50 model for deep feature extraction. - F(·): Fully connected layers combining features for classification. - Optimizer: Adam optimizer with a learning rate of 0.001. - Loss: Binary Crossentropy loss function.3: Output    - Trained dual-stream model for fingerprint liveness detection.4: Begin5: Feature Extraction    - Extract features from training images:     - FVGG = VGG16(Xtrain)F_{VGG} = VGG16(X_{train})FVGG = VGG16(Xtrain).    - FResNet = ResNet50(Xtrain)F_{ResNet} = ResNet50(X_{train})FResNet = ResNet50(Xtrain).6: Features Fusion    - Concatenate the features:    - Fcombined = [FVGG,FResNet]F_{combined} = [F_{VGG}, F_{ResNet}]Fcombined = [FVGG,FResNet].7: Model Architecture    - Define fully connected layers:    - Layer 1: Dense layer with 256 neurons and ReLU activation.    - Layer 2: Dense layer with 128 neurons and ReLU activation.    - Layer 3: Dense output layer with 1 neuron and Sigmoid activation.8: Compilation    - Compile the model using Adam optimizer and Binary Crossentropy loss.9: Training    - Train the model using FcombinedF_{combined}Fcombined and YtrainY_{train}Ytrain:    - Batch size: 32    - Epochs: 50    - Validation split: 10%10: Evaluation    - Evaluate on test dataset for performance metrics (accuracy, precision, recall, F1-score).11: End Procedure

## 3. Experimental Setup

This section covers the various databases used for our experimental evaluation, the evaluation’s performance measures, and the specifics of our method’s implementation.

### 3.1. Database

The proposed model’s effectiveness is assessed using two publicly available datasets, i.e., LivDet2013 and LivDet2015 [6,21]. Four different partitions of the LivDet 2015 data-base, which was first adopted in the fourth edition of fingerprint liveness detection, are used to validate the suggested model’s overall performance. The databases include Bio-metrika, Green Bit, Digital Persona, and CrossMatch. Each database has its own training and testing fingerprint samples. More details about different partitions of LiveDet 2015 are available in Table 1 and Table 2. LivDet2013 also contains two datasets, which are, respectively, Biometrika and ItalData. Further information regarding the various partitions of LivDet2013 can be found in Table 3. The dataset release agreement and download links for all LivDet databases are available at: https://livdet.org/registration.php (2 February 2025).

These databases are extensively utilized in fingerprint spoof detection research, owing to their comprehensive and diverse datasets. The databases feature a wide range of spoof materials and sensor types, which make them a robust foundation for assessing the performance of spoof detection systems. Their widespread adoption ensures that results derived from these datasets are both relevant and comparable to existing studies in the field.

### 3.2. Performance Metrics

In this work, both the Attack Presentation Classification Error Rate (APCER), which indicates the percentage of misclassified spoof fingerprint images, and the Bonafide Presentation Classification Error Rate (BPCER), which reflects the error rate for genuine fingerprint images, were used to assess classification accuracy. APCER and BPCER are represented by Equations (1) and (2), respectively:

(1) $$APCER=\frac{Number\;of\;incorrectly\;classified\;fake\;samples}{Total\;number\;of\;fake\;samples}\times 100$$

(2) $$BPCER=\frac{Number\;of\;incorrectly\;classified\;live\;samples}{Total\;number\;of\;live\;samples}\times 100$$

Moreover, the Average Classification Error (ACE) is calculated as the average of APCER and BPCER. Equation (3) describes the formulation of ACE:

(3) $$ACE=\frac{APCER+BPCER}{2}$$

The ACE is also used to calculate the accuracy of the proposed model, as formulated in Equation (4).

(4) Accuracy=100−ACE

## 4. Experimental Results and Comparative Evaluation

In this work, a variety of experiments were conducted to determine spoofing at-tempts that could compromise fingerprint verification systems. Moreover, the live and fake fingerprint training samples must be shuffled before being input into the network. In all experiments, the model parameters were initialized using Gaussian distributions, with the weights randomly sampled from a distribution with a fixed mean and standard devi-ation. Furthermore, it should be underlined that fingerprint labels are required; a live fin-gerprint has the label “1”, whereas a false fingerprint has the label “0”. There are two types of models: real fingerprints and fake fingerprints. The model’s final output, which is known as the anticipated probability, is converted into the predicted label based on the threshold when a fingerprint sample enters the network. Consequently, the prediction is accurate if the expected value matches the actual label; however, if not, it is considered in-correct.

### 4.1. Experimental Results and Comparative Evaluation on LiveDet 2013 Database

**Figure 3:**
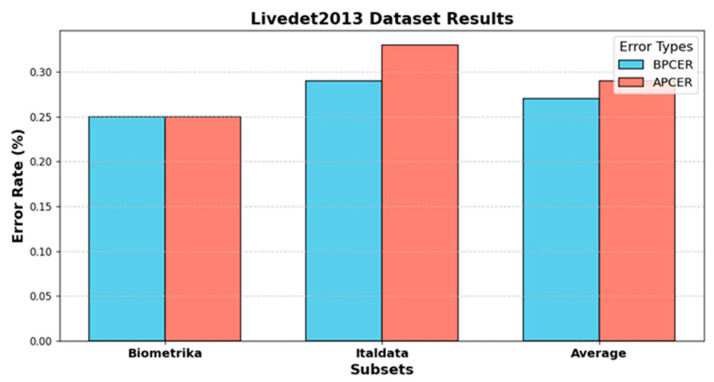
BPCER and APCER error rates for Biometrika and Italdata subsets in the Livedet2013 dataset.

The results presented in Table 4 and Table 5, along with Figure 3, highlight the performance and error rates of different liveness detection methodologies on the Livedet2013 dataset. Table 5 shows that the proposed method achieves the highest average accuracy of 99.72% across the Biometrika and Italdata subsets. This outperforms other methods like the Gram model (98.95%) and Pre-trained CNN (98.45%). This indicates a significant improvement over earlier techniques, such as BP-ANN, Improved DCNN, and DRBM + DBM, which display lower accuracies, especially on the Italdata subset. Table 4 and Figure 3 provide error rate analyses, highlighting a balanced BPCER and APCER of 0.25% for the Biometrika subset. In contrast, for the Italdata subset, APCER reaches 0.33%, slightly higher than BPCER at 0.29%. This suggests that Italdata may contain more challenging spoof patterns. These patterns could affect the model’s performance in differentiating between bona fide and spoof fingerprints. The average ACE of 0.28% across subsets shows that although the state-of-the-art methods maintain reasonable error rates, the proposed method’s exceptional performance demonstrates enhanced robustness and reduced error rates. By combining VGG16 and ResNet50, our method leverages the detailed feature extraction of VGG16 and the hierarchical deep feature learning of ResNet50. This combination can capture a broader range of features, from fine details to complex patterns, enhancing overall accuracy. Other methods like the Gram model or Pre-trained CNN might rely on single architectures that do not capture the full range of features as effectively. For example, a single CNN might miss some hierarchical features that ResNet50 can capture. This highlights the effectiveness of the proposed methodology in accurately identifying genuine and spoof fingerprints across diverse data subsets.

### 4.2. Experimental Results and Comparative Evaluation on LiveDet 2015 Database

**Figure 4:**
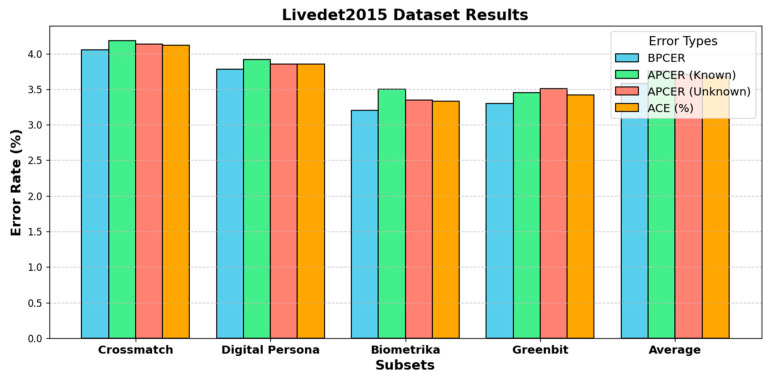
BPCER and APCER error rates for Biometrika and Italdata subsets in the Livedet 2015 dataset.

The outcomes from Table 6 and Table 7, and Figure 4 highlight the performance and error rates of various liveness detection methodologies on the Livedet2015 dataset. Table 6 pre-sents error rates for four subsets (i.e., Crossmatch, Digital Persona, Biometrika, and Green-bit) across different metrics, i.e., BPCER, APCER (known and unknown spoof materials), and ACE. Among these subsets, Crossmatch exhibits the highest error rates with an ACE of 4.12%, but Biometrika shows the lowest error rate with an ACE of 3.33%. The average ACE across subsets is 3.68%, suggesting reasonable performance overall. However, varia-tions in spoof materials, especially unknown materials, introduce challenges, as reflected in the slightly higher APCER rates.

Table 7 compares the accuracy of various liveness detection methodologies across the same subsets. While the Gram model achieves the highest accuracy on the Crossmatch subset (i.e., 99.63%), its performance drops significantly on Digital Persona (i.e., 91.5%), indicating sensor-specific challenges. The proposed method achieved an average accuracy of 96.32%, demonstrating balanced performance across all subsets. Notably, it performs exceptionally well on Biometrika (96.67%) and Greenbit (96.58%), highlighting its strong generalization capability. In contrast, the state-of-the-art methods like RF classifier and LivDet 2015 show lower average accuracies of 95.78% and 95.39%, respectively, while DLTP, which lacks data on individual subsets, has a notably lower average accuracy of 86.39%. In Table 7, the LFLDNet [16] method achieved the highest average accuracy (97.80%) on the LivDet 2015 dataset. However, it is important to note that this method utilized CycleGAN to generate synthetic forged fingerprint images, thereby augmenting the dataset with additional samples of Ecoflex and gelatin materials. This augmentation significantly enhanced the generalization ability of their model by incorporating diverse and challenging training data. In contrast, our proposed method was trained and evaluated only on the original LivDet 2015 dataset, without any additional synthetic data. Despite this limitation, our method achieved a competitive average accuracy of 96.32%. It outperformed most methods trained and tested on the original standard dataset. The only method that surpassed ours was LFLDNet and did so by leveraging an enhanced dataset, which is not part of the standard LivDet 2015 evaluation protocol. Therefore, our method’s performance highlights its robustness and effectiveness, particularly within the constraints of the original/standard dataset.

Figure 4 illustrates error rates (i.e., BPCER, APCER Known, APCER Unknown, and ACE) for each subset. It shows that BPCER rates are generally lower than APCER rates for unknown materials, which is consistent with Table 6 data. The higher APCER values for unknown spoof materials in Crossmatch and Greenbit imply these subsets may have more challenging spoof variations. Overall, the proposed method’s balanced accuracy and lower error rates across known and unknown spoof materials underline its robustness. However, the variability in APCER highlights areas for further improvement, especially with unknown spoof challenges.

## 5. Conclusions

This paper presents a dual pre-trained model that integrates VGG16 and ResNet50 for fingerprint liveness detection. The approach capitalizes the strengths of both architectures; i.e., VGG16 is utilized for high-resolution feature extraction, while ResNet50 excels in capturing deeper and more complex patterns. This approach is evaluated on the Live-det2013 and Livedet2015 datasets, achieving impressive accuracy levels. On Livedet2013, the proposed method attains an accuracy of 99.72%, outperforming existing models like the Gram model and BP-ANN. On Livedet2015, it maintains a strong performance with an average accuracy of 96.32% that shows its robustness across various subsets and spoof materials. The error rate analysis reveals balanced BPCER and APCER scores. However, APCER values are slightly higher for unknown spoof types, particularly in the Cross-match subset of Livedet2015, indicating potential areas for further optimization. Overall, the dual-model approach effectively enhances generalization and accuracy and is well-suited for diverse liveness detection scenarios. In future work, we aim to enhance the framework’s robustness against adversarial attacks [30] by exploring defensive strategies, ensure its resilience in real-world security scenarios. Moreover, we plan to improve the scheme’s performance on unknown spoof types and study its capability for real-time deployment on resource-constrained devices, such as embedded systems or mobile platforms. Additionally, expanding its evaluation across newer datasets and sensor technologies will further validate its applicability and versatility.

## Figures and Tables

**Table 1:** Summary of the three Liveness Detection (LivDet2015) datasets used in this study (Digital Persona, Green Bit, and Biometrika).

Dataset	Live (Train/Test)	Spoof (Train/Test)	Spoofing Materials					
			Ecoflex	Gelatine	Latex	WoodGlue	Liquid Ecoflex	RTV
**Digital Persona**	1000/1000	1000/1500	250	250	250	250	250	250
**Green Bit**	1000/1000	1000/1500	250	250	250	250	250	250
**Biometrika**	1000/1000	1000/1500	250	250	250	250	250	250

**Table 2:** Summary of the CrossMatch partition of Liveness Detection (LivDet2015) datasets used in this study.

Dataset	Live (Train/Test)	Spoof (Train/Test)	Spoofing Materials				
			Body Double	Ecoflex	Playdoh	OOMOO	Gelatin
**CrossMatch**	1510/1500	1473/1448	300	270	281	297	300

**Table 3:** Summary of the two Liveness Detection (LivDet2013) datasets used in this study (ItalData and Biometrika).

Dataset	Live (Train/Test)	Spoof (Train/Test)	Spoofing Materials				
			Ecoflex	Gelatine	Latex	WoodGlue	Modasil
**ItalData**	1000/1000	1000/1000	200	200	200	200	200
**Biometrika**	1000/1000	1000/1000	200	200	200	200	200

**Table 4:** Error rates (BPCER, APCER, ACE) for different subsets of the Livedet2013 dataset.

Dataset	Subsets	BPCER (%)	APCER (%)	ACE (%)
Livedet2013	**Biometrika**	0.25	0.25	0.25
	**Italdata**	0.29	0.33	0.31
	**Averag**	0.27	0.29	0.28

**Table 5:** Accuracy (%) comparison of various liveness detection methodologies on Livedet2013 dataset.

Liveness Detection Methodology	Accuracy (%)		
	Biometrika	Italdata	Average
Gram model [22]	99.15	98.75	98.95
BP-ANN [23]	96.45	97.65	97.05
Improved DCNN [24]	95.65	98.6	97.12
Pre-trainedCNN [7]	99.20	97.7	98.45
TP/LM CNN [25]	94.12	97.92	96.02
DRBM + DBM [26]	96.00	94.50	95.25
LFLDNet [16]	99.25	99.80	99.52
**Proposed Method**	**99.75**	**99.69**	**99.72**

**Table 6:** Error rates (BPCER, APCER, ACE) for different subsets of the Livedet2015 dataset.

Dataset	Subsets	BPCER	APCER (Known)	APCER (Unknown)	ACE (%)
Livedet2015	**Crossmatch**	4.05	4.18	4.13	4.12
	**Digital Persona**	3.78	3.92	3.85	3.85
	**Biometrika**	3.20	3.50	3.35	3.33
	**Greenbit**	3.30	3.45	3.51	3.42
	**Average**	3.58	3.76	3.71	3.68

**Table 7:** Accuracy (%) comparison of various liveness detection methodologies on Livedet2015 dataset.

Liveness Detection Methodology	Accuracy				
	Crossmatch	Biometrika	Digital Persona	Greenbit	Average
Gram model [22]	99.63	95.9	91.5	97.30	96.08
RF classifier [27]	98.07	95.22	94.16	95.7	95.78
CNN [28]	98.60	95.80	90.50	96.20	95.27
LivDet 2015 [6]	98.10	94.36	93.72	95.40	95.39
LFLDNet [16]	97.28	98.68	96.44	98.64	97.8
DRBM+DBM [26]	95.00	-	-	-	95.00
DLTP [29]	-	-	-	-	86.39
**Proposed Method**	**95.88**	**96.67**	**96.15**	**96.58**	**96.32**

## Data Availability

The original data used for training the models in this study are available on: https://livdet.org/registration.php.
